# CLC channel function and dysfunction in health and disease

**DOI:** 10.3389/fphys.2014.00378

**Published:** 2014-10-07

**Authors:** Gabriel Stölting, Martin Fischer, Christoph Fahlke

**Affiliations:** ^1^Institute of Complex Systems-Zelluläre Biophysik (ICS-4), Forschungszentrum JülichJülich, Germany; ^2^Institut für Neurophysiologie, Medizinische Hochschule HannoverHannover, Germany

**Keywords:** CLC channel, anion channel, patch clamp, myotonia congenita, Bartter syndrome, leukencephalopathy

## Abstract

CLC channels and transporters are expressed in most tissues and fulfill diverse functions. There are four human CLC channels, ClC-1, ClC-2, ClC-Ka, and ClC-Kb, and five CLC transporters, ClC-3 through −7. Some of the CLC channels additionally associate with accessory subunits. Whereas barttin is mandatory for the functional expression of ClC-K, GlialCam is a facultative subunit of ClC-2 which modifies gating and thus increases the functional variability within the CLC family. Isoform-specific ion conduction and gating properties optimize distinct CLC channels for their cellular tasks. ClC-1 preferentially conducts at negative voltages, and the resulting inward rectification provides a large resting chloride conductance without interference with the muscle action potential. Exclusive opening at voltages negative to the chloride reversal potential allows for ClC-2 to regulate intracellular chloride concentrations. ClC-Ka and ClC-Kb are equally suited for inward and outward currents to support transcellular chloride fluxes. Every human CLC channel gene has been linked to a genetic disease, and studying these mutations has provided much information about the physiological roles and the molecular basis of CLC channel function. Mutations in the gene encoding ClC-1 cause myotonia congenita, a disease characterized by sarcolemmal hyperexcitability and muscle stiffness. Loss-of-function of ClC-Kb/barttin channels impairs NaCl resorption in the limb of Henle and causes hyponatriaemia, hypovolemia and hypotension in patients suffering from Bartter syndrome. Mutations in *CLCN2* were found in patients with CNS disorders but the functional role of this isoform is still not understood. Recent links between ClC-1 and epilepsy and ClC-Ka and heart failure suggested novel cellular functions of these proteins. This review aims to survey the knowledge about physiological and pathophysiological functions of human CLC channels in the light of recent discoveries from biophysical, physiological, and genetic studies.

## Introduction

**Figure 1:**
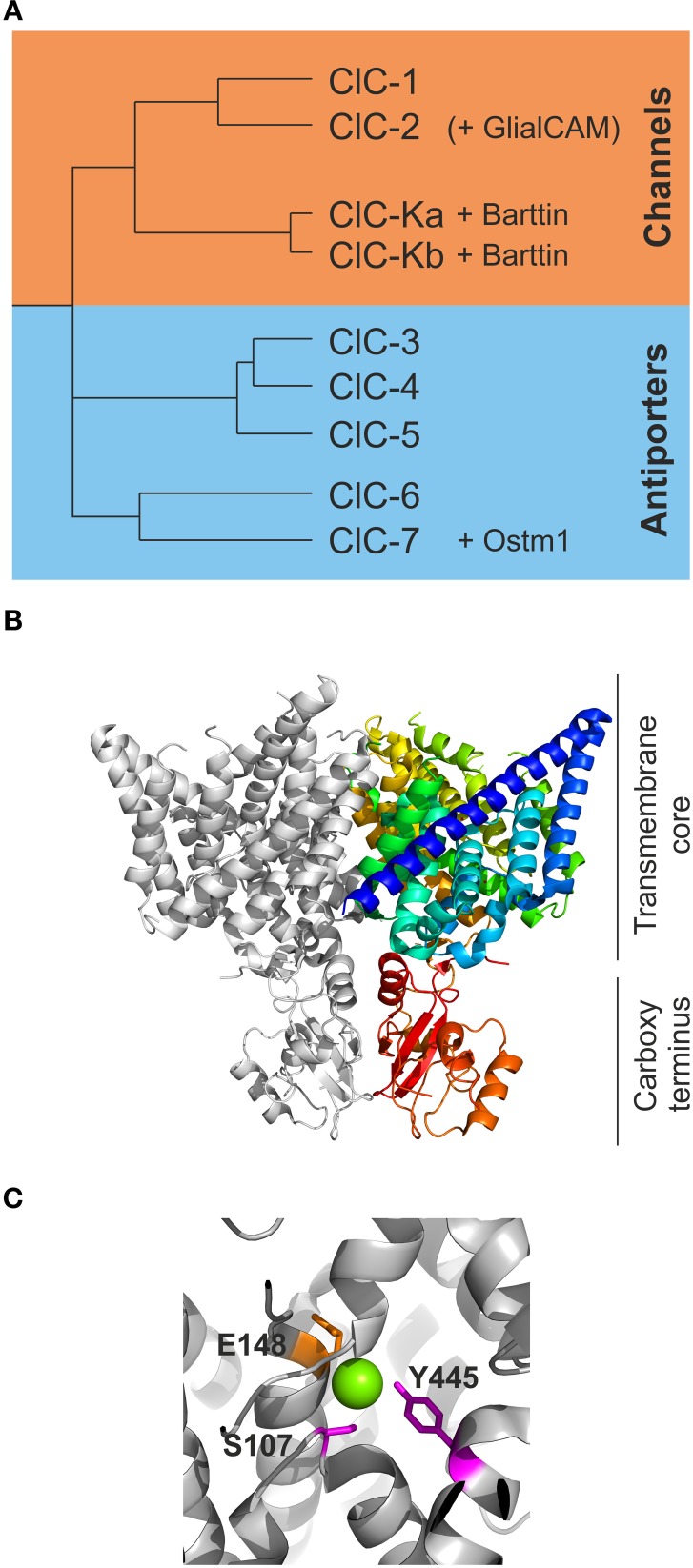
**The CLC family of transport proteins encompasses chloride channels and chloride/proton exchangers. (A)** A phylogram demonstrates the early separation of the human CLC proteins into one branch of chloride channels encompassing ClC-1, ClC-2, ClC-Ka, and ClC-Kb. The chloride/proton antiporters encompass ClC-3 through −7. ClC-K channels require the subunit barttin while ClC-7 is dependent on the presence of Ostm1 for normal function. Recently, GlialCAM has been identified as accessory subunit of ClC-2. **(B)** A view of the dimer of cmClC [PDB ID: 3ORG (Feng et al., 2010)] in a ribbon presentation. One subunit is shown in light gray while the other subunit is shown in color ranging from the beginning of the B-helix in blue to the carboxy terminus in red. The upper part of the protein comprises the transmembrane core while the lower red part contains the carboxy termini with the CBS domains. **(C)** A closer view of some of the critical residues coordinating the chloride ions in ecClC (PDB ID: 1OTS). The magenta colored residues Y445 and S107 are responsible for the binding of chloride (green sphere) in S_cen_ and are also proposed to be critical for slow gating of the CLC channels. E148 represents the so-called “gating glutamate” swinging from S_cen_ to S_ext_ and is thought to be tightly involved in fast protopore gating of CLC channels.

Expression cloning of a chloride channel from *Torpedo marmorata*—now called ClC-0—by Jentsch et al. (1990) established the CLC family of anion transport proteins (Figure 1A). The CLC family encompasses nine human CLC proteins, ClC-1 through −7 and the ClC-Ka and -Kb proteins. Single-channel recordings on ClC-0 revealed unitary conductances of about 10 pS (Miller, 1982), and since this value predicts a transport rate far above the maximum rates of transporters and pumps there was little doubt that this protein mediates anion diffusion through an aqueous conduction pathway. Unitary current amplitudes of ClC-1 and ClC-2 supported this idea (Pusch et al., 1994; Weinreich and Jentsch, 2001), so that all members of the family were initially assumed to represent anion channels. It thus came as a surprise when a bacterial homolog in *E. coli* was shown to function as a coupled anion/proton exchanger (Accardi and Miller, 2004). Successive analysis of transport mechanisms of human CLC isoforms demonstrated that five out of nine human CLCs, ClC-3 through ClC-7, represent anion-proton exchangers rather than anion channels (Picollo and Pusch, 2005; Scheel et al., 2005; Neagoe et al., 2010; Leisle et al., 2011; Guzman et al., 2013). The CLC family thus combines two functional groups with thermodynamically different transport processes. All experimental data presented so far support a close structural similarity of the members of the CLC family and demonstrate that transport proteins of similar structure can specialize into ion channels or transporters.

CLC channels and transporters fulfill different physiological tasks. Whereas CLC exchangers are mainly expressed in intracellular compartments such as endosomes or lysosomes and seem to contribute to housekeeping regulation of these organelles, CLC channels are located in the surface membrane of excitable and epithelial cells and contribute to the regulation of membrane excitability as well as to the transport of electrolytes, water, and nutrients. In this review, we focus exclusively on the channel branch of the CLC family, summarizing their function, their cellular roles and possible involvement in diseases.

## Molecular physiology of CLC channels

### Structural determinants of CLC channel function

So far, no high-resolution three-dimensional structure has been reported for a CLC channel. However, CLC anion/proton exchangers have been crystallized from several prokaryotic and eukaryotic species (Dutzler et al., 2002; Accardi et al., 2006; Lobet and Dutzler, 2006; Feng et al., 2010; Robertson et al., 2010; Jayaram et al., 2011; Lim et al., 2012). Since structural properties of CLC transporters translate well for structure-function investigations performed on CLC channels (Miller, 2003), it is generally assumed that CLC channels are structurally very similar to CLC transporters.

All CLC proteins are assembled as dimers, with each subunit exhibiting 18 transmembrane helices (named A to R) followed by a cytosolic carboxy-terminus that contains two conserved cystathionine-ß-synthase (CBS) domains in eukaryotic CLCs (Bateman, 1997) (Figure 1B). Each subunit binds anions without any contribution from the other subunits—in full agreement with two separate active centers that transport anions independently in the two neighboring subunits. This peculiar architecture had already been suggested based on early single channel recordings that demonstrated two equally spaced conductance states and suggested the existence of two ion conduction pathways, the so-called protopores, per individual CLC channel (Miller, 1982; Miller and White, 1984). Three separate chloride binding sites within each subunit were identified in the three-dimensional structures, S_int_, S_cen_, and S_ext_, named after their position relative to the extra- or intra-cellular side of the plasma membrane. The central position forms a significant part of the selectivity filter with the conserved amino acids S107, I109, F357, and Y445 in addition to the less well conserved F348 and I356 (Figure 1C; all positions given for EcClC) coordinating the chloride ion within the conduction pathway (Dutzler et al., 2002, 2003; Lobet and Dutzler, 2006). There are no full positive charges such as from arginine or lysine side chains involved in the formation of anion binding sites, in agreement with the notion that CLC proteins use helical dipole moments to generate a positive electrostatic potential necessary for anion selectivity (Dutzler et al., 2002; Gouaux and MacKinnon, 2005).

The separate CLC transporter structures show a similar backbone fold, but appear to represent different conformations of the Cl^−^/H^+^ exchange cycle. Comparison of these conformations reveals movements of a conserved glutamate side chain, E148 in EcClC (Figure 1C), from inside to outside of the conduction pathway switching from S_cen_ to S_ext_. Such movements depend on the protonation status and likely account for the transport of protons in a 2 Cl^−^ to 1 H^+^ stoichiometry (Dutzler et al., 2003; Accardi and Miller, 2004; Feng et al., 2010; Picollo et al., 2012).

The two carboxy-terminal CBS domains of eukaryotic CLC channels are known to interact and form intra-molecular dimeric complexes. Partial removal of CBS domains can cause either loss-of-function, or alterations in channel gating or subcellular distribution (Maduke et al., 1998; Estévez et al., 2004; Hebeisen et al., 2004; Hebeisen and Fahlke, 2005; Garcia-Olivares et al., 2008). A recent structure of a eukaryotic CLC transporter suggested interactions between CBS domains and the intracellular poles of helices D and R. Since these helices contain the serine and tyrosine side chains coordinating S_cen_ (S107 and Y445 in EcClC) this interaction provides a structural basis for the regulation of CLC function by clarboxy-terminal domains (Hebeisen and Fahlke, 2005; Feng et al., 2010). Additional inter-subunit interactions of CBS dimers was observed *in vitro* for isolated ClC-5 and ClC-Kb carboxy-termini (Meyer et al., 2007; Martinez and Maduke, 2008). Such interactions might play a role in inter-subunit communication and might contribute to cooperative gating processes (Bykova et al., 2006).

### Functional properties of CLC channels in voltage clamp experiments

**Figure 2:**
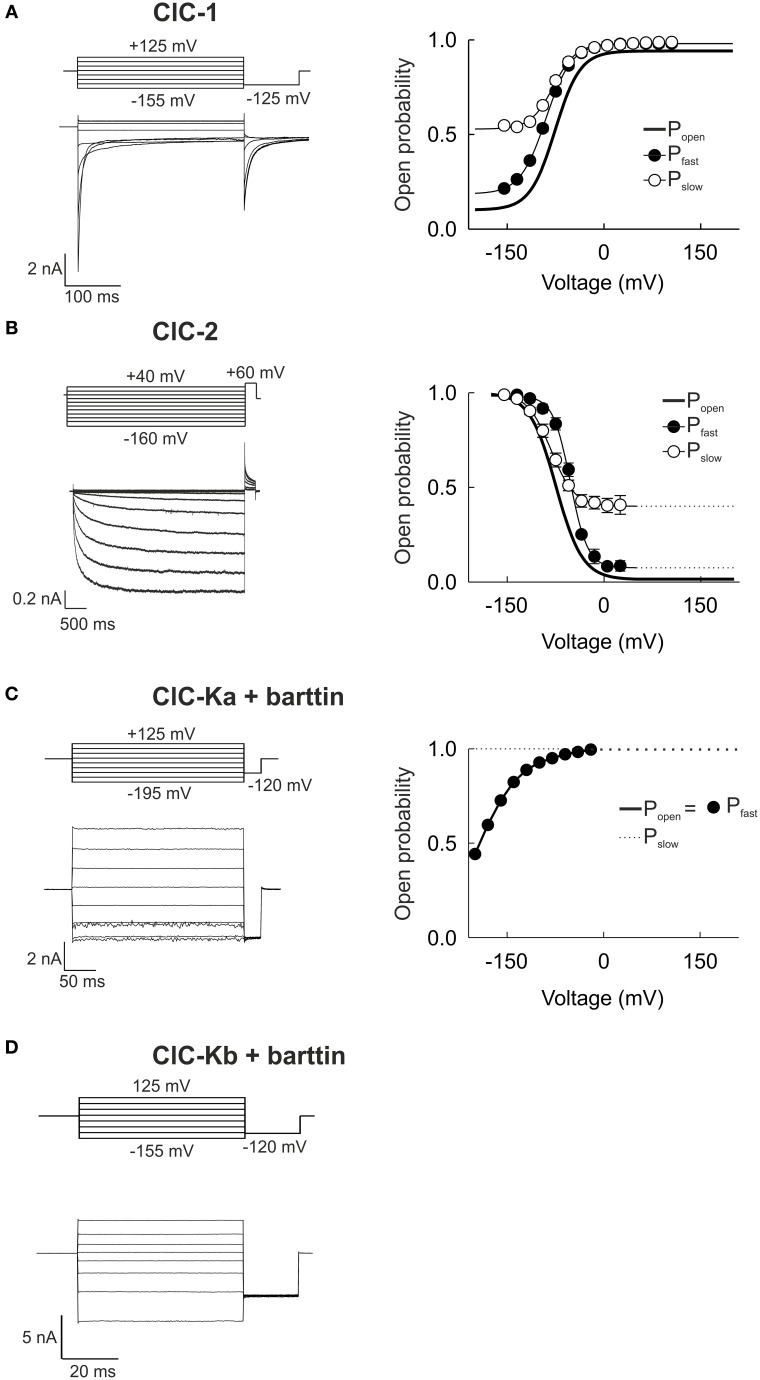
**Whole-cell patch clamp recordings demonstrate the functional variability of human CLC channels. (A–D)** Representative whole-cell current responses from HEK293T cells expressing ClC-1, ClC-2, ClC-Ka/barttin, or ClC-Kb/barttin to the indicated voltage protocols (left column). Right column shows the voltage dependences of the open probabilities of fast protopore (filled circles) and slow common gates (open circles) as well as of the probability of the channel to be conductive (thick line). The ClC-1 recording is reproduced from Weinberger et al. (2012) while ClC-Ka and −Kb recordings were reproduced from Riazuddin et al. (2009).

Heterologous expression of CLC channels in *Xenopus* oocytes and mammalian cells allowed detailed functional analysis of all human CLC channels. Figure 2 shows whole-cell patch-clamp recordings from HEK293 cells over-expressing human CLC channels and illustrates the functional diversity of these distinct proteins. For all recordings, cells were subject to a nearly symmetrical distribution of chloride across the plasma membrane, held at 0 mV and voltage steps over a broad range were applied.

Whereas ClC-Ka/barttin and ClC-Kb/barttin current amplitudes are almost time-independent, voltage steps result in time- and voltage-dependent relaxations of the ClC-1 and ClC-2 current amplitude that can be used for the kinetic analysis of channel gating. Changes in macroscopic current amplitude usually follow the sum of two exponential functions with separate time constants. The two activation and deactivation time constants are due to the existence of two gating mechanisms that either act on separate protopores or jointly on both protopore (Miller, 1982). In all mammalian CLC channels protopore gating is about 10-fold faster than common gating (Miller, 1982; Saviane et al., 1999; Fischer et al., 2010; Stölting et al., 2013) which led to the synonymous use of the terms fast/protopore gate and slow/common gate. For many channels these kinetic differences were used to separate the two processes in macroscopic current recordings. The instantaneous current amplitude at a tail pulse of fixed voltage translates into the relative open probability of the channel at the end of the preceding test voltage (Hodgkin and Huxley, 1952). Inserting a short test pulse to a voltage at which the two gating processes differ sufficiently in speed only manipulates the fast gate and thus effectively fixes it at the same open probability for all preceding voltage steps. Such an approach allows for measurements of the voltage dependence of the slow gating process in isolation. On the assumption that the fast and slow gating processes are independent, the open probability of the fast gate can be calculated as the ratio of the total open probability of the channel to the percentage of open slow gates (Accardi and Pusch, 2000).

The skeletal muscle chloride channel ClC-1 (Figure 2A) is open at 0 mV, with fast and slow gates that are both activated by membrane depolarization (Accardi and Pusch, 2000). Plotting the current amplitude vs. the membrane potential reveals an inwardly rectifying conductance (Fahlke et al., 1995). The ubiquitously expressed ClC-2 is closed at positive potentials and activates on a very slow time course upon membrane hyperpolarization (Figure 2B) (De Santiago et al., 2005). ClC-2 gating is again characterized by two kinetically different gating processes that are both activated by hyperpolarization rather than by depolarization as in ClC-1. Both ClC-1 and ClC-2 display strongly inward rectifying macroscopic current amplitudes, but with a different mechanistic basis. Inward rectification of ClC-1 channels is due to voltage-dependent unitary conductances that decrease at positive voltages to approximately 10% of the conductance at negative potentials (Pusch et al., 1994; Fahlke et al., 1995; Rychkov et al., 1998; Stölting et al., 2014). In contrast, ClC-2 unitary current conductances are voltage-independent, and rectification of ClC-2 currents is due to voltage-dependent gating that closes the fast gate at voltages positive to 0 mV (Stölting et al., 2013).

ClC-Ka (Figure 2C) and ClC-Kb (Figure 2D) can only be functionally expressed together with the accessory subunit barttin (Estévez et al., 2001; Waldegger et al., 2002; Scholl et al., 2006; Fischer et al., 2010). In mammalian cells, ClC-Kb/barttin currents are time-independent with slight bidirectional rectification. In contrast, ClC-Ka/barttin channels close very rapidly upon steps to voltages negative to −100 mV. These very fast processes likely correspond to fast protopore gating in these channels (Riazuddin et al., 2009; Fischer et al., 2010). When expressed in oocytes, ClC-Ka/barttin and ClC-Kb/barttin display dramatically different gating properties (Imbrici et al., 2014). Whereas absolute open probabilities of ClC-Ka/barttin are close to 1 in mammalian cells (Riazuddin et al., 2009), corresponding values are very small in *Xenopus* oocytes (Gradogna et al., 2010). Moreover, ClC-K channels expressed in mammalian cells often respond to pharmacological modification in a different way than channels expressed in *Xenopus* oocytes (Imbrici et al., 2014). The reason for these functional differences is not yet clear. There might be yet unknown interaction partners of ClC-K/barttin that are present in one expression system, but not in the other one. Alternatively, the functional properties of ClC-K/barttin channels might depend on the lipid composition. Although expression in mammalian cells appear to be more similar to renal cells than amphibian oocytes it is not possible to judge at the moment which of the two distinct biophysical and pharmacological phenotypes is closer to the functional properties of native channels.

Chloride is by far the most abundant anion *in vivo* thus also leading to the designation of CLC channels as “chloride” instead of “anion” channels. One must note that the selectivity filter of CLC channels does not perfectly distinguish between different kinds of anions seen as a permeability for other (unphysiological) anions such as I^−^, F^−^, SCN^−^, NO^−^_3_, or Br^−^. However, studies using the other abundant physiological anion, HCO^−^_3_, are rare. A significant permeability for bicarbonate was found in studies of ClC-5 in *Xenopus laevis* oocytes (Mo et al., 1999) and a mathematical study of epithelial ion fluxes in the ascending limb of Henle predicts that a significant bicarbonate conductance of ClC-Kb is necessary for proper renal function (Weinstein, 2010).

### Double barreled CLC channels exhibit unique single channel properties

**Figure 3:**
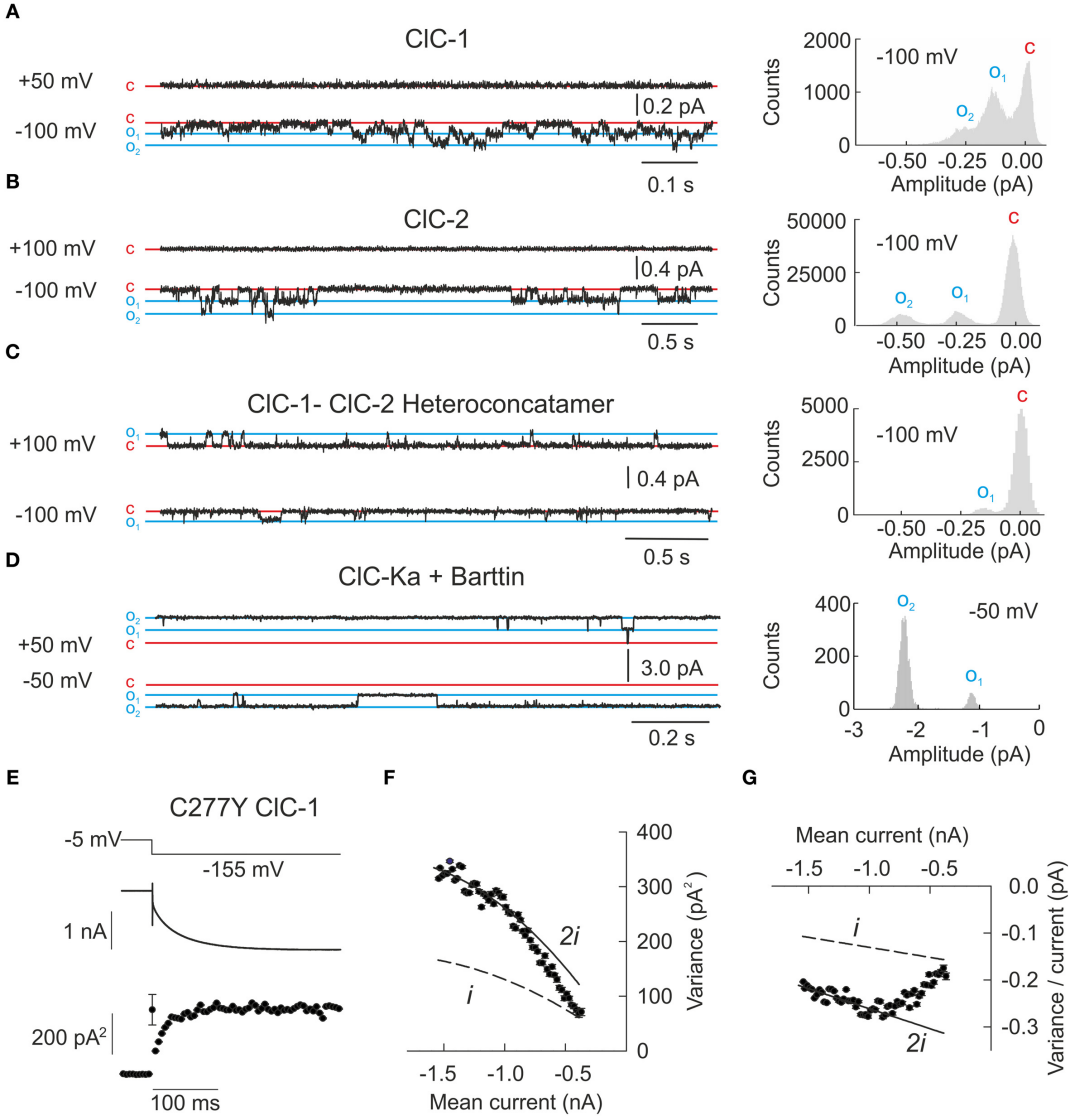
**Single channel recordings or noise analysis of whole cell recordings provide properties of individual CLC channels. (A–D)** Representative single channel recordings from homodimeric ClC-1, homodimeric ClC-2, heteroconcatameric ClC-1-ClC-2 as well as ClC-Ka co-expressed with barttin. All recordings from homodimeric channels show two separate open states representing one or two simultaneously open protopores. ClC-Ka/barttin exhibits a high open probability approaching 1 so few deviations from the fully opened state are seen. **(A–C)** were reproduced and modified from Stölting et al. (2014) and **(D)** from Riazuddin et al. (2009). **(E)** Representative plot of the mean current and variance of HEK293T cells expressing the human ClC-1 channel with cysteine 277 exchanged to tyrosine. Voltage protocol (top), mean current (middle), and variance (bottom) are obtained at pH 5.9 to increase the open probability of channels containing this particular mutant. **(F)** Plotting variance against the mean current does not result in a simple parabolic distribution. As expected for double barreled channels with distinct protopore and cooperative gating all data points fall in between the theoretical prediction for channels with permanently open common gate (denoted as “i”) or permanently open protopore gate (denoted as “2i”). The transition from one parabola (dashed line) to the other one (line) indicates the slow transition of the fast gate from closed to open over the recording time. **(G)** Linear transformation of the plot in **(F)** facilitates the identification of the changes to fast gate open probability. **(E–G)** were reproduced and modified from Weinberger et al. (2012).

Single channel recordings now exist for the majority of human CLC channels (Figures 3A–D) (Saviane et al., 1999; Fischer et al., 2010; Weinberger et al., 2012; Stölting et al., 2013). When analyzing recordings from membrane patches that contain only one CLC channel the amplitude histogram plot therefore shows a total of three peaks, corresponding to the closed channel, or to the opening of either one or two protopores (right side of Figures 3A–D). This property results from the unique dimeric architecture of CLC channels with two ion conduction pathways.

Single channel recordings from the prototypic ClC-0 revealed long periods with closed channels that were interrupted by phases in which both protopores rapidly switch between open and closed states. During these burst-like phases, the distribution of the probabilities of encountering any of the three amplitude levels is determined almost exclusively by the fast protopore gate whereas the long closed durations are based on the slow common gate. Since individual gating of protopores is independent of the neighboring subunit, the times spent in each of the three current levels is binomially distributed and this behavior has been termed “binomial bursts” (Miller, 1982). ClC-1 and ClC-2 exhibit gating processes that resemble the behavior of ClC-0, with fast protopore gating and slow cooperative processes (Figures 3A,B) (Saviane et al., 1999; Accardi and Pusch, 2000; Zúñiga et al., 2004). Barttin appears to lock the cooperative gate in an open conformation, so that single channel recordings of ClC-Ka/barttin channels exclusively show an even number of independently gated protopores (Fischer et al., 2010). No single channel recordings in heterologous expression systems have been reported so far for ClC-Kb.

The analysis of heterodimeric channels consisting of one ClC-1 and one ClC-2 subunit provided novel insights into the molecular basis of slow cooperative gating (Stölting et al., 2014). Such hetero-dimeric channels lack cooperative gating but retain two separate fast and slow gating mechanisms for each protopore. In ClC-1-ClC-2 hetero-dimers, fast protopore gating of ClC-1 resembles corresponding processes in ClC-1 homodimers. However, the ClC-1 protopore is kept closed at positive potentials by a novel slow gate that permits only transient openings in the hetero-dimeric assembly. In contrast, the ClC-2 protopore is active at all potentials and is under control of fast and slow gating processes that are kinetically different from those identified for the ClC-1 protopore. In single channel recordings, this behavior resulted in only one conductance state corresponding to the ClC-2 protopore (Figure 3C). These results suggest that common gating of CLC channels ultimately arises from conformational changes within an individual protopore (Bennetts and Parker, 2013; Stölting et al., 2014). Homo-dimerization permits the synchronization of these processes and common gating. In hetero-dimers this coordination is impaired so that slow gating steps are not cooperative anymore.

Single channel amplitudes for the ClC-2 protopore were significantly smaller in hetero-concatamers than in ClC-2 homo-dimers (Figure 3C). Taking together, these experiments demonstrate how closely the two subunits cooperate within one individual channel. Gating and permeation through every transport protein is governed by electrostatic interactions between charged side chains or helical dipole moments. One could imagine that changes in the orientation of defined side chains or of helices via inter-subunit interactions might adjust the electrostatic field of the adjacent CLC subunit and thus cooperatively determine gating and permeation.

Noise analysis is an alternative technique for determining the properties of individual channels (Sigworth, 1980). It is particularly useful for channels with small unitary current amplitude and has often helped correlating unitary and macroscopic currents of certain ion channels. Such analysis is based on the assumption that the underlying gating processes are stochastic events and that small deviations in macroscopic current amplitude are caused by fluctuations in the number of open channels. Whereas current noise generated by channels with only one conductance levels is easy to analyze, the double-barreled architecture of CLC channels requires modifications of such noise analysis (Fischer et al., 2010; Weinberger et al., 2012).

Voltage steps cause a relaxation of the open probability of voltage-dependent channels from the previous equilibrium value to the new one and result in time-dependent changes of the current amplitude. During these current relaxations the single channel current amplitude is constant whereas the number of open channels changes. Repetitive sampling of current responses to the same voltage step (Figure 3E) permits determination of the mean current over all recorded voltage steps and mean variances for multiple time points after the voltage step. For channels with only one conductance state a plot of the current variance (σ^2^) vs. the mean amplitude <*I*> (Figure 3F) yields a parabolic distribution that depends on the: single channel amplitude (*i*) and the number of channels (*N*).

$$\sigma^{2}=\;i\langle I\rangle -\left(\frac{{\langle I\rangle }^{2}}{N}\right)$$

or after linearization by dividing the variance by current

$$\frac{\sigma^{2}}{\langle I\rangle }=i-\frac{\langle I\rangle }{N}$$

Some CLC channels show a significant deviation from this theory, which is even clearer after linearization of the parabolic plot (Figures 3F,G). The basis for this peculiar current-variance relationship is the double-barreled architecture of these channels. Taking two protopores with two structurally and kinetically distinct gating processes permits a satisfactory quantification of this behavior. The macroscopic current of the CLC channels is given by the product of the open probability of each gate, the number of channels and twice the single protopore amplitude:

$$I=N\;\cdot 2i\;\cdot P_{p}(t)\cdot P_{c}(t)$$

The current variance depends on the number of channels (*N*), the single protopore amplitude (*i*) and the time-dependent open probabilities for the fast protopore gate (*P_p_*(*t*)) and the slow common gate (*P_c_*(*t*)):

$$\sigma^{2}=N\;\cdot 2i^{2}\;\cdot \;P_{p}(t)\cdot P_{c}(t)\cdot \left(1-2P_{p}\left(t\right)\cdot P_{c}\left(t\right)+P_{p}\left(t\right)\right)$$

These equations can be simplified to:

$$\sigma^{2}=\;(1+P_{p}(t))\cdot i\langle I\rangle -\left(\frac{{\langle I\rangle }^{2}}{N}\right)$$

demonstrating that this quantitative treatment permits the single channel amplitude to be determined if one of the two open probabilities is known. Even if the variance-current plot does not show obvious deviations from a parabolic shape, the dependence of the initial slope on the minimum open probability of the protopore gate (*P_p_*) has to be taken into account.

In all cases current variances must fall in between two extremes that are given by:

$$\begin{array}{l} \sigma^{2}=2i\cdot \langle I\rangle -\left(\;\frac{{\langle I\rangle }^{2}}{N}\right)\text{ for }P_{p}(t)=1\\ \sigma^{2}=i\cdot \langle I\rangle -\left(\;\frac{{\langle I\rangle }^{2}}{2N}\right)\text{ for }P_{c}(t)=1 \end{array}$$

Using such an analysis we demonstrated that a myotonia-causing mutation, C277Y, results in a significant decrease of the slow gate open probability (Weinberger et al., 2012). Assuming a maximal value of twice the lowest determined variance/current ratio, these results also demonstrated diminished single channel amplitudes as compared to wild type ClC-1 from single channel recordings. This finding is rather unexpected since C277 does not contribute to the formation of the conduction pathway in the known three-dimensional structures. It provides additional support for the notion of a tight interaction of subunits in determining the gating and permeation properties of CLC channels.

### Molecular determinants of CLC channel function

In the past 20 years the combination of site-directed mutagenesis and functional analysis of mutant channels has provided insights into mechanisms and sequence determinants of CLC channel gating and anion permeation. Site-directed mutagenesis experiments on several CLC channels demonstrated that the same conserved glutamate at the beginning of the F helix whose movement is necessary for proton transport in CLC antiporters is important for fast gating in ClC-0, ClC-1, and ClC-2 (Fahlke et al., 1997; Dutzler et al., 2003; Niemeyer et al., 2003; De Santiago et al., 2005) and was therefore named “gating glutamate.” In ClC-K channels this “gating glutamate” is replaced by valine, and ClC-Ka and ClC-Kb are therefore often assumed to lack protopore gating. However, single channel recordings from ClC-Ka/barttin revealed short gating events and a very high open probability of the channel (Riazuddin et al., 2009; Fischer et al., 2010). The time spent in the open and closed states was found to be binomially distributed and the highest observed conductance level was always a multiple of two demonstrating the observed opening and closing transitions are caused by individual protopore gating without interference by the permanently open common gate (Riazuddin et al., 2009; Fischer et al., 2010). The homologous rat ClC-K1 is also functional in the absence of barttin and thus provides the opportunity to study the effects of barttin on ClC-K function in more detail. In the absence of barttin, rClC-K1 displays protopore and common gating processes with opposite voltage dependence. Co-expression with barttin results in permanent opening of the common gate, again as shown by the binomial distribution of the amplitude histograms for each conductance state upon co-expression with barttin (Fischer et al., 2010). Although the results imply that side chains separate from the original “gating glutamate” must be involved in protopore gating, the precise molecular basis of such gating has not yet been identified in ClC-K channels.

In ClC-0, ClC-1, and ClC-2 a cysteine close to the interface domain and in close proximity to S_ext_ (C212 in ClC-0, C277 in ClC-1 or C258 in ClC-2) is critically involved in slow cooperative gating (Lin et al., 1999; Accardi et al., 2001; Zúñiga et al., 2004; De Santiago et al., 2005; Weinberger et al., 2012). Again, ClC-K channels lack a cysteine at this position suggesting that this side chain is not required for common gating. Based on the three-dimensional structures of CLC antiporters, it was recently proposed that slow cooperative gating of ClC-0 and ClC-1 depends on the tyrosine residue that coordinates the central chloride binding site (Bennetts and Parker, 2013). This tyrosine is believed to interact with the gating glutamate as the final step of slow gating to occlude or open the pore. How the common gate is simultaneously conferred to both protopores is, however, still unknown. It is not even known if the antiporters within the CLC family are even under control of a cooperative gating mechanism. Based on previous studies it seems reasonable, however, that rearrangements of the carboxy-terminus might be required for cooperative gating (Bykova et al., 2006; Garcia-Olivares et al., 2008; Ma et al., 2011; Stölting et al., 2013).

### Conversion of CLC antiporters into channels/uniporter

To understand the structural features that distinguish CLC anion channel and anion-proton exchanger attempts were undergone to convert CLC antiporters into anion channels or vice versa. However, these attempts have not been fully successful so far. The substitution of a glutamate residue (“gating glutamate” E148 in EcClC) within the F helix by alanine abolishes secondary-active transport and permits passive anion permeation (Accardi and Miller, 2004; Jayaram et al., 2008). The corresponding exchange also abolished the coupling of proton transport to chloride flux in the human anion/proton antiporters ClC-4 and ClC-5 (Picollo and Pusch, 2005; Scheel et al., 2005). Another glutamate residue in EcClC (E203) could also be neutralized by substitution with an alanine to impair the coupling of chloride and proton transport resulting in a switch toward a channel-like phenotype possibly due to a lack of the internal binding site for protons (Accardi et al., 2005). Some exchanges of the central tyrosine (Y445 in EcClC) also resulted in an apparent channel phenotype with a strong decrease of the observed anion density for S_cen_ in accompanying X-ray crystal structures (Accardi et al., 2006). Mutating E148 together with Y445 increased unitary anion transport in EcClC above values observed for uniporters, however, the observed conduction rates were well below corresponding values in CLC channels (Jayaram et al., 2008). It should be noted, however, that all of these residues are mostly conserved even across the channel branch of the CLC family thus providing important insight into the conduction process of anions and protons but failing to demonstrate the actual difference between transporters and channels from the same family. There are no reports of a CLC channel being converted into an antiporter so far.

## Cellular physiology and pathophysiology of CLC channels

### CLC-1

Adult skeletal muscle is unique among excitable tissues in its high resting chloride conductance. It exceeds the sarcolemmal potassium conductance by a factor of more than four and is so high that changes in the extracellular chloride concentrations do not modify the resting potential, but rather results in changes of the intracellular [Cl^−^] until the equilibrium potential of chloride ions again equals the muscle resting potential (Hodgkin and Horowicz, 1959; Bretag, 1987). The physiological role of ClC-1 became clear by studying the pathophysiology of myotonia congenita, a rare human condition that is characterized by muscle stiffness upon sudden forceful movements (Bryant and Morales-Aguilera, 1971; Adrian and Bryant, 1974). In myotonic muscle fibers, the membrane potential does not completely repolarize after a series of action potentials during voluntary contractions, resulting in the so-called after-depolarization. After-depolarizations can result in prolonged electrical activity of the muscle fibers even after the end of neuronal activity and are thus the electrophysiological basis to muscle stiffness (Adrian and Bryant, 1974). The time course of the action potential itself is unchanged in muscle fibers from the myotonic goat indicating that ClC-1 does not contribute to the repolarization of the action potential itself (Bryant, 1973).

**Figure 4:**
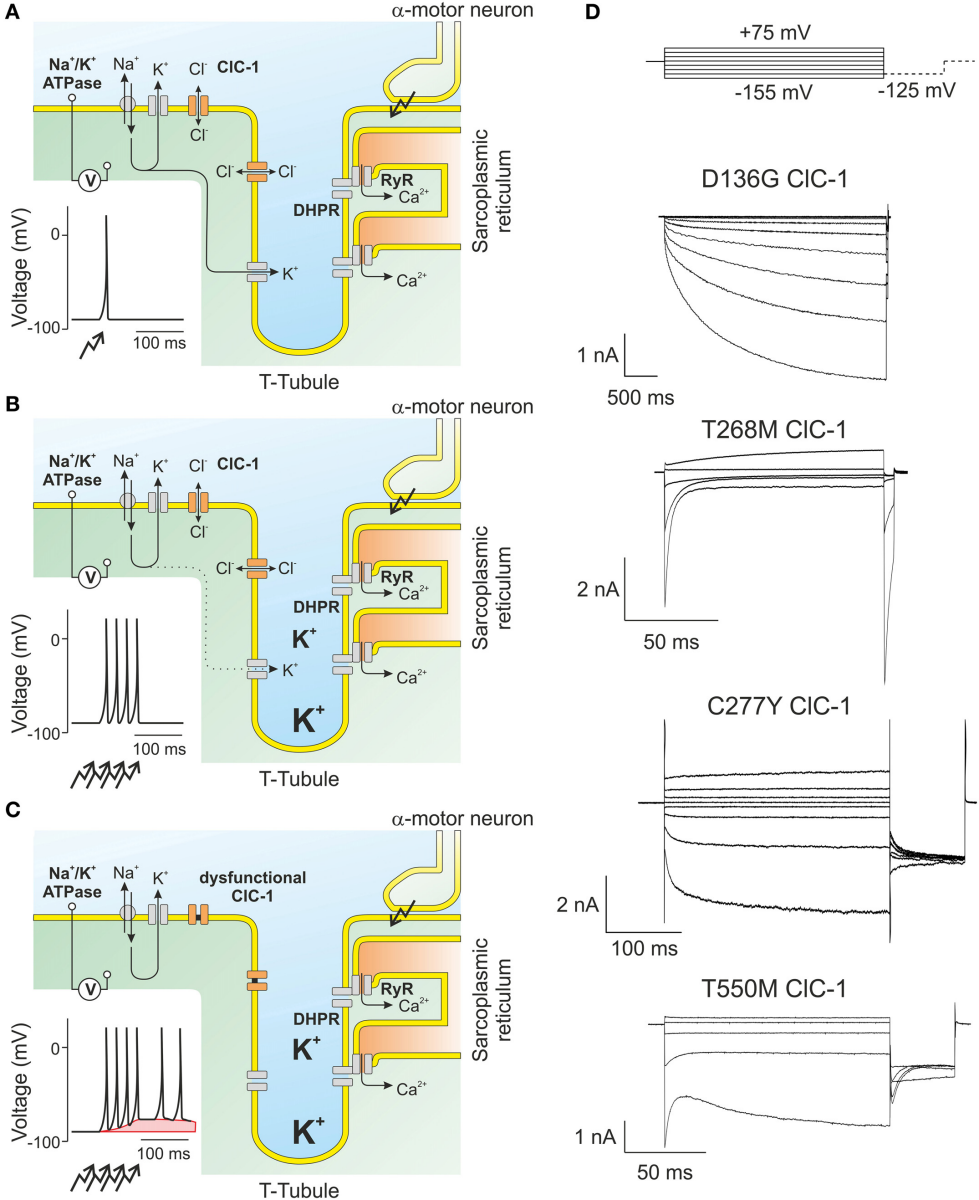
**ClC-1 is the major muscle chloride channel. (A)** In skeletal muscle, the resting membrane potential is determined by the potassium gradient across the sarcolemmal and t-tubular membrane. Action potentials results in the opening of L-type calcium channels (DHPR) that in turn open intracellular channels (RyR) releasing calcium from the sarcoplasmic reticulum needed for the contraction of the muscle. **(B)** A train of action potentials results in the displacement of potassium through channels to the extracellular side. As the diffusion of ions from the t-tubules is slow, potassium accumulates and causes transient changes of the potassium reversal potential which are, however, offset by the high sarcolemmal chloride conductance. **(C)** In muscle fibers expressing dysfunctional ClC-1 channels, t-tubular depolarization is propagated to the surface membrane and can trigger spontaneous generation of new action potentials even after the end of the voluntary movement. This “after-depolarization” is marked in red. **(D)** Representative recordings from mutant ClC-1 channels carrying disease-causing mutations illustrating the diverse results of single amino acid exchanges on ClC-1 gating.

The after-depolarization of myotonic muscle fibers is due to the accumulation of K^+^ within the t-tubules that occurs in normal as well as in myotonic muscle during repetitive action potentials. In normal skeletal muscle, high resting chloride conductance reduces the sarcolemmal length constant and prevents propagation of this depolarization along the sarcolemma. In the absence of the shunting sarcolemmal chloride conductance in myotonic muscle, the t-tubular depolarization leads to depolarization of the sarcolemmal membrane causing sustained activity of the muscle fiber. It is still under debate whether ClC-1 mediates its shunting conductance only through channels localized along the outer sarcolemmal membrane or along the t-tubules as well. A recent study presented evidence for a homogeneous distribution across t-tubules and the sarcolemmal membrane (DiFranco et al., 2011) while another paper described an exclusive expression in the surface membrane (Lueck et al., 2010). Regardless of the exact localization, muscle chloride channels allow skeletal muscle fibers to electrically tolerate t-tubular membrane invaginations and the subsequent potassium accumulation (Figures 4A–C).

ClC-1 was cloned from skeletal muscle as the first mammalian member of the CLC family (Steinmeyer et al., 1991). Mutations in *CLCN1* as the genetic basis of myotonia congenita proved that ClC-1 indeed represents the adult skeletal muscle chloride channel (Koch et al., 1992; George et al., 1993). ClC-1 exhibits an inwardly rectifying unitary current conductance at symmetrical chloride concentrations with a value of approximately 1.5 pS below −85 mV and a 10-fold lower conductance at voltages positive to the chloride reversal potential (Pusch et al., 1994; Stölting et al., 2014). ClC-1 exhibits an open probability of 0.38 at −85 mV (Weinberger et al., 2012) and only opens minimally during the upstroke of the action potential, which is faster than the ClC-1 activation time course (Fahlke and Rüdel, 1995; Accardi and Pusch, 2000; Hebeisen and Fahlke, 2005). These specific anion conduction and gating properties make ClC-1 ideally suited for providing a large resting chloride conductance with only minimum interference with the action potential. These features minimize Na^+^ influx during the action potential and thus reduce ATP consumption following sustained muscular activity that is required for active Na^+^ extrusion via the Na^+^/K^+^-ATPase.

Although voltage-dependent gating of ClC-1 is not strictly necessary for its physiological function, almost all disease-causing mutations that have been studied perturb channel gating [with the notable exception of G230E that affects ion permeation and selectivity of ClC-1 (Fahlke et al., 1997)]. The first myotonia mutation that was functionally analyzed, D136G (Figure 4D), couples channel opening to the intracellular chloride concentration and thus inverts the voltage dependence of ClC-1 (Fahlke et al., 1995). D136G hClC-1 only opens at voltages below the chloride equilibrium potential. Chloride efflux at such voltages will reduce the intracellular chloride concentration until reaching a chloride reversal potential similar to the sarcolemmal resting potential. At these [Cl^−^] D136G hClC-1 will be permanently closed, in full agreement with low resting chloride conductance and hyperexcitability. D136G hClC-1 resembles ClC-2 in many properties and thus illustrates the importance of isoform-specific specialization within the CLC family. Subsequently, several mutations were identified that shifted the activation curve of ClC-1 to more depolarized potentials (Pusch et al., 1995; Rhodes et al., 1999; Wu et al., 2002) (Figure 4D). Such shifts decrease the open probability of hClC-1 at −85 mV and thus decrease resting chloride conductance. A similar change in channel gating is the basis of myotonia in the goat (Beck et al., 1996). Other mutations result in changes of gating so that the two gating processes develop an opposing pattern of voltage-dependence, one stimulated by membrane depolarization and the other by hyperpolarization as in T550M ClC-1 channels (Warnstedt et al., 2002; Wu et al., 2002; Weinberger et al., 2012). In the last two decades a large variety of disease-causing mutations have been found in patients with myotonia congenita. These mutations are spread over the whole coding region of ClC-1, and their functional analysis has provided many important insights into sequence determinants of CLC channel function (Fahlke et al., 1995, 1997; Wollnik et al., 1997; Saviane et al., 1999; Richman et al., 2012; Weinberger et al., 2012; Lee et al., 2013).

Recently, a novel ClC-1 variant was identified by exomic sequencing in patients suffering from idiopathic epilepsy (Chen et al., 2013). This study also showed ClC-1 mRNA transcripts and protein bands stained with antibodies against ClC-1 in parts of the human brain. This novel role and localization of ClC-1 might lead to new perspectives for the physiology of CLC channels in the central nervous system. However, the absence of central neurological symptoms in patients with myotonia congenita or in animal models of this disease raises the question of how functional changes in ClC-1 might contribute to epilepsy.

### CLC-2

ClC-2 was discovered shortly after ClC-1 in excitable as well as in non-excitable cells (Thiemann et al., 1992). ClC-2 exhibits a voltage-independent single channel conductance of only ~2.4 pS per protopore (Stölting et al., 2013). It is closed at positive potentials and activates over a very slow time course upon hyperpolarization. After activation at negative potentials, voltage steps back to positive potentials result in a very slow current deactivation. Whereas the protopore gate closes completely at positive potentials the slow common gate exhibits a minimum open probability well above zero (De Santiago et al., 2005; Garcia-Olivares et al., 2008). Similar gating features have been observed in *Xenopus* oocytes and in mammalian cells, however, activation and deactivation kinetics were significantly slower in oocytes. The kinetics of gating is modified by nucleotide binding to the carboxy-terminus (Dhani et al., 2008; Stölting et al., 2013) and by the surrounding lipid composition (Hinzpeter et al., 2007). It was also found that the activation and deactivation gating of ClC-2 is significantly accelerated by deletions or modifications of carboxy- or amino-terminal domains suggesting the involvement of large rearrangements of these protein domains (Garcia-Olivares et al., 2008; Saint-Martin et al., 2009; Stölting et al., 2013) such as reported for ClC-0 and ClC-1 (Bykova et al., 2006; Ma et al., 2011).

Gating of ClC-2 depends on intracellular [Cl^−^] so that channel opening only occurs at potentials negative to the Cl^−^ equilibrium potential (Niemeyer et al., 2004). In cells in which the membrane potential is clamped by channels of different selectivity to more negative potentials it might be assumed that ClC-2 will stay open and permit anion efflux until the chloride equilibrium potential equals the membrane potential. ClC-2 could therefore contribute to the regulation of intracellular anion concentrations in neurons, glia, or to the efflux of chloride in epithelial cells. Early studies on ClC-2 heterologously expressed in *Xenopus* oocytes revealed activation by osmotic gradients, suggesting that ClC-2 might be involved in cellular osmotic homeostasis (Gründer et al., 1992). However, functional properties of ClC-2 are clearly different from volume-activated anion channels (Nilius et al., 1997), and cells lacking ClC-2 exhibit unchanged volume-activated anion currents (Nehrke et al., 2002).

ClC-2 can assemble with accessory subunits that are not required for the function of ClC-2, but rather determine the subcellular localization and modify channel gating in different cell types. The first reported accessory subunit of ClC-2 was the protein cereblon which binds to the carboxy-terminus of ClC-2 and putatively regulates the expression of functional channels in the retina (Hohberger and Enz, 2009). More recently, a novel subunit called GlialCAM (synonymous with the earlier designation HepaCAM) has been shown to associate with ClC-2 (Jeworutzki et al., 2012). GlialCAM is a protein of 416 amino acids in length with one transmembrane domain and a large Ig-like V- and C2-type amino terminal domain. In heterologous expression systems, co-expression of GlialCAM with ClC-2 switched the phenotype toward an almost constitutively open channel. While GlialCAM is only interacting with ClC-2 *in vivo* due to its cell specific expression, it was found to associate with multiple CLC channels *in vitro*, such as ClC-0, ClC-1, and ClC-K/barttin affecting localization and common gating parameters (Jeworutzki et al., 2014).

ClC-2 currents recorded from brain slices from GlialCAM knock-out animals are not dramatically different from corresponding traces obtained from WT animals, suggesting that GlialCAM does not exert the same dramatic effect on channel gating in native cells as in heterologous expression system (Hoegg-Beiler et al., 2014). Work on MLC1 knock-out mice furthermore suggested that MLC1, a membrane protein of unknown function associated to megalencephalic leukoencephalopathy (Leegwater et al., 2001), might stabilize GlialCAM in the plasma membrane and thus act as an additional binding partner within the ClC-2/GlialCAM complex (Hoegg-Beiler et al., 2014).

**Figure 5:**
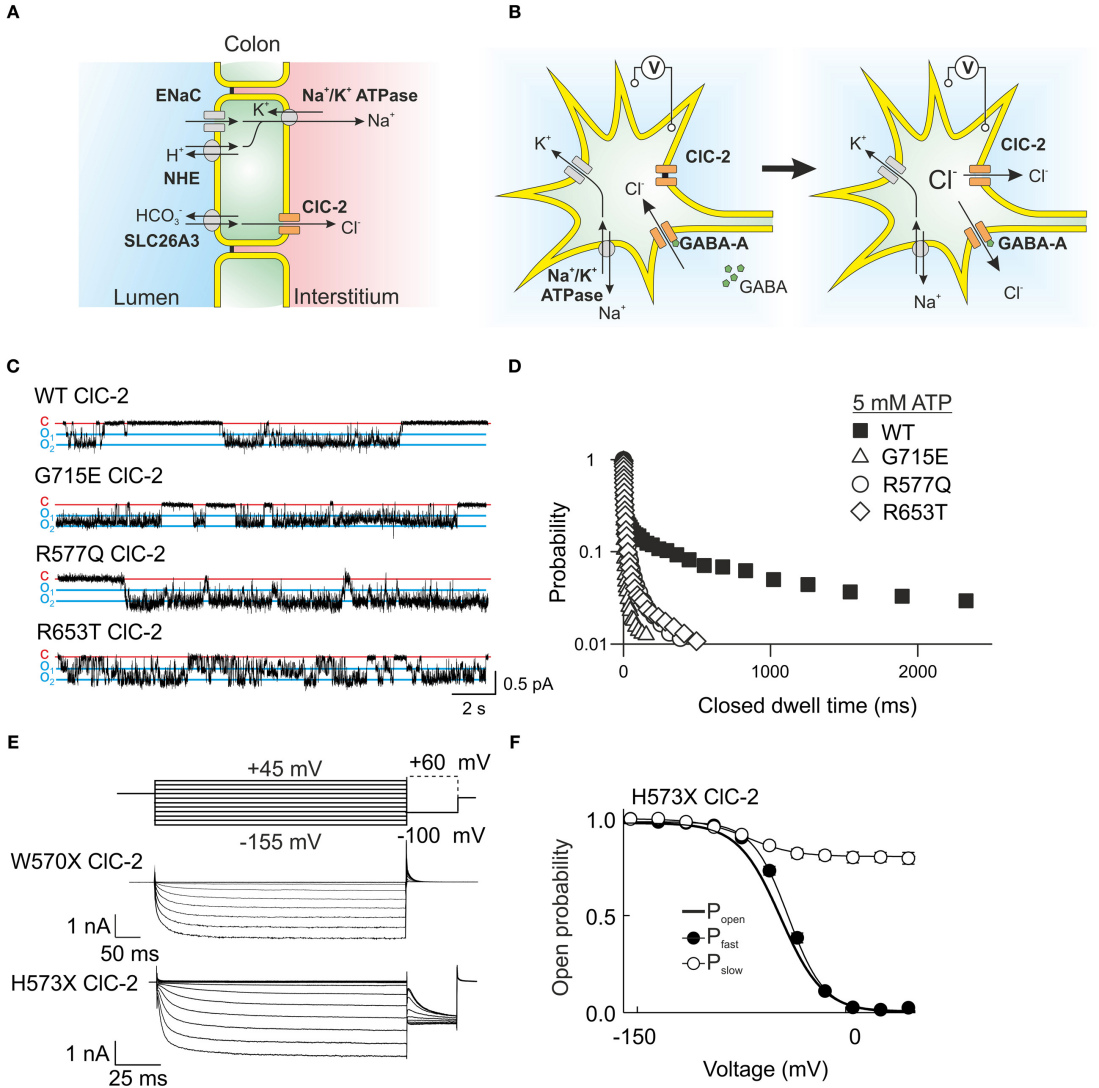
**ClC-2 is expressed in epithelia as well as excitable cells. (A)** In colonic enterocytes chloride is absorbed from the luminal side via a chloride/bicarbonate exchanger and then transported through basolateral ClC-2 to the interstitium. Sodium follows through a luminal channel or sodium/proton exchanger and is transported to the interstitium via the Na^+^/K^+^ ATPase (Catalán et al., 2002). **(B)** The role of ClC-2 in excitable cells such as neurons is still under debate. A probable mechanism leaves ClC-2 closed at the potassium equivalent resting membrane potential but changes in the chloride reversal potential through Cl^−^ influx open ClC-2 and permit chloride efflux through this channel. Chloride efflux possibly causes membrane depolarization and hyperexcitability. **(C)** Single channel recordings from mutant ClC-2 channels with mutations found in patients with idiopathic epilepsies. These mutations shorten the long closed states caused by closures of the common gate. **(D)** A plot of the probability finding the ClC-2 channel in the closed state for the indicated duration demonstrates the disease-associated changes of common. **(E)** W570X—that was recently found in patients with idiopathic generalized epilepsy—causes similar acceleration of ClC-2 activation and deactivation as the previously studied mutant H573X when expressed in HEK293 cells. **(F)** H573X partially opens the slow gate of ClC-2. **(C,D)** were reproduced and modified from Stölting et al. (2013). The data from H573X ClC-2 was reproduced and modified from Garcia-Olivares et al. (2008).

Among the best understood roles of ClC-2 is the chloride efflux at the basolateral membrane in the gastro-intestinal tract, facilitating NaCl and subsequently H_2_O reabsorption (Figure 5A) (Catalán et al., 2002, 2004). A putative activator of ClC-2, lubiprostone, ameliorates chronic idiopathic constipation, probably by stimulating the secretion of chloride and water into the colon lumen. The success of this treatment is in agreement with a role of ClC-2 also in apical chloride secretion (Cuppoletti et al., 2004). During development, ClC-2 is highly expressed in lung tissue and has been proposed as a potential pathway to replace the missing chloride conductance in patients suffering from cystic fibrosis (Schwiebert et al., 1998, −2). However, *Clcn*2^−/−^ mice do not suffer from lung disease, and additional ClC-2 knock-out in *CFTR*^−/−^ mice did not deteriorate the phenotype of cystic fibrosis these animals (Zdebik et al., 2004). Studies of knock-out mice also revealed a testicular degeneration and retinal degeneration, underlining the roles of ClC-2 in retinal pigment epithelia and in Sertoli cells where this channel might be involved in regulating the highly specialized fluid secretion of these tissues (Bösl et al., 2001). Although not linked to any renal disease, ClC-2 was also early on shown to be expressed in the kidney (Thiemann et al., 1992). An analysis of mRNA transcripts revealed a significant expression in all nephron segments except for the cortical collecting duct and the outer medullary collecting duct and was found to be modulated by aldosterone in rats (Ornellas et al., 2002). ClC-2 was also shown to be expressed in skeletal muscle (Thiemann et al., 1992). However, ClC-2 currents could neither be recorded from kidneys nor skeletal muscle. It is conceivable that ClC-2 might form heterodimers in these tissues where the other CLC channels such as ClC-1 (Lorenz et al., 1996; Stölting et al., 2014) or the ClC-K channels are expressed.

ClC-2 is expressed in neuronal and glial cells (Staley, 1994; Nobile et al., 2000; Sìk et al., 2000), but its role in the central nervous system is still not sufficiently understood. Mutations in *CLCN2* were found in human patients with idiopathic generalized epilepsy as well as in leukoencephalopathy (D'Agostino et al., 2004; Kleefuß-Lie et al., 2009; Saint-Martin et al., 2009; Klassen et al., 2011; Depienne et al., 2013; Stölting et al., 2013). *CLCN2* loss-of-function mutations result in myelin vacuolation in the brain and spinal cord and in mild neurological deficits such as cerebellar ataxia (Depienne et al., 2013). The lack of ClC-2 cause similar pathological changes in human patients as observed in *Clcn*2^−/−^ mice. Similar changes in brain morphology are caused by the loss of GlialCam and MLC1, most likely because these proteins assemble into a protein complex that prevents the formation of such vacuoles.

*Clcn*2^−/−^ mice display abnormal cortical activity and a higher susceptibility to induced seizures, but do not show overt signs of epileptic seizures animals (Blanz et al., 2007; Cortez et al., 2010). Changes in the dynamic regulation of intraneuronal chloride concentrations might be the basis of this hyperexcitability (Blanz et al., 2007; Földy et al., 2010). Activation of GABA_A_ receptors hyperpolarizes neurons via a transient chloride influx. ClC-2 might be activated by the resulting increases in intracellular [Cl^−^] and provide an exit pathway for chloride ions (Figure 5B). This mechanism was first demonstrated in experiments in which ClC-2 was expressed in dorsal root ganglion cells. This maneuver reduced [Cl^−^]_int_ and inverted depolarizing into hyperpolarizing GABA currents (Staley et al., 1996). Another possible mechanism postulates that ClC-2 channels might remain opened at positive voltages and permit a repolarizing influx of chloride (Ratté and Prescott, 2011).

Several *CLCN2* missense mutations found in patients suffering from idiopathic epilepsies change ClC-2 gating in a similar fashion (Figures 5C,D), i.e., faster activation and deactivation time courses. This uniform functional consequence supports the notion that these changes in channel gating might contribute to the pathogenesis of epilepsy. In mutant ClC-2, efflux of chloride after repetitive GABA stimulation will activate more rapidly and thus conduct a depolarizing current with more rapid onset than WT channels. A causal role of *CLCN2* mutations has been questioned in the past by several authors (Niemeyer et al., 2010; Depienne et al., 2013; Jentsch, 2013). One argument was that these mutations are not linked to epilepsy in a Mendelian fashion. Some of these sequence variations display incomplete co-segregation and occur not only in affected, but also in unaffected individuals of the same family. However, such inheritance pattern is not uncommon in idiopathic generalized epilepsy. There is increasing evidence that this disease is not simply due to the occurrence of single disease-causing mutations, but rather results from the co-existence of multiple sequence variations affecting distinct genes (Klassen et al., 2011). The manifestation and the severity of the disease depend on the combined functional consequences of multiple coincident genetic risk factors favoring hyperexcitability in the central nervous system. A polygenic heterogeneity model suggests that *CLCN2* mutations affect neuronal excitability but that these changes only result in epilepsy together with mutations in other genes (Klassen et al., 2011).

One of the mutations linked to idiopathic epilepsy, W570X, was also identified in a genome sequencing study of leukoencephalopathy patients (Depienne et al., 2013). This mutation is very similar to a truncation of ClC-2 studied earlier in our laboratory and causes a strong acceleration of gating activation and inactivation with an almost constitutively open slow gate (Figures 5E,F) (Garcia-Olivares et al., 2008). However, the patient suffering from leukoencephalopathy did not show seizures, putatively due to almost complete degradation of the truncated mutant channel (Depienne et al., 2013).

### CLC-Ka/CLC-Kb

**Figure 6:**
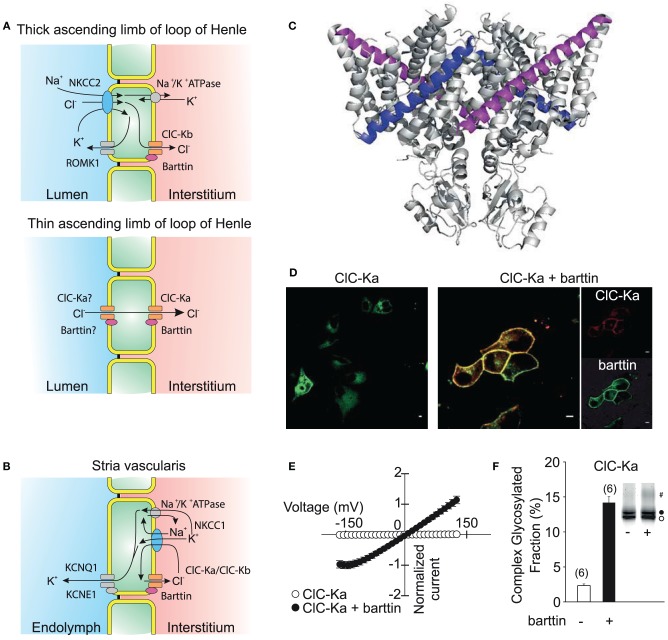
**ClC-K channels are necessary for transepithelial solute transport in the loop of Henle and the stria vascularis of the inner ear. (A)** Expression of ClC-Ka/barttin and ClC-Kb/barttin in the thin ascending and thick ascending limb of the loop of Henle. Chloride is absorbed on the luminal side either by a secondary-active transport mechanism or diffusion through apical channels and then conducted through ClC-Ka/barttin or ClC-Kb/barttin to the interstitial side. ClC-Ka/barttin is necessary for the passive reabsorption of NaCl in the thin ascending limb. In the thick ascending limb, ClC-Kb/barttin supports the basolateral chloride efflux that is necessary for the electrogenic NaCl absorption. The absorption of NaCl establishes a transepithelial potential that additionally drives the paracellular flux of Mg^2+^ or Ca^2+^. **(B)** ClC-Ka/barttin and ClC-Kb/barttin mediate basolateral chloride efflux and support charging the endolymph via K^+^ secretion within the stria vascularis. **(C)** Barttin is thought to bind to the B (magenta) and J (blue) helix of ClC-K channels (Tajima et al., 2007). **(D)** Confocal fluorescence images (kindly provided by Dr. Daniel Wojciechowski) of YFP-ClC-Ka alone show a predominant staining of intracellular membranes (left side). Upon co-expression of CFP-barttin, ClC-Ka is relocalized to the plasma membrane (right side). **(E)** Barttin switches ClC-Ka and ClC-Kb into an active state as seen in a normalized current vs. voltage plot. **(F)** Barttin increases complex glycosylation (#) and the stability of the ClC-K/barttin complex in the plasma membrane. **(A,B)** are modified after Fahlke and Fischer (2010).

ClC-Ka and ClC-Kb are exclusively expressed in the nephron and in the stria vascularis of the inner ear (Figures 6A,B). The physiological role of these proteins was clarified by the generation of the *Clcnk*1^−/−^ mouse (ClC-K1 is the rodent homolog of ClC-Ka) with pronounced *diabetes insipidus* and the linkage of *CLCNKB* to Bartter syndrome, a human condition characterized by impaired renal urine concentration resulting in hypotension with elevated renin and aldosterone levels (Simon et al., 1997; Matsumura et al., 1999). These findings suggested that ClC-Ka and ClC-Kb are crucial for normal urinary concentration.

Initial attempts to characterize the function of these channels failed (Kieferle et al., 1994) since ClC-Ka and Kb can only be functionally expressed together with the accessory subunit barttin (Estévez et al., 2001; Waldegger et al., 2002; Scholl et al., 2006). Barttin was cloned as the gene product of the disease gene of Bartter syndrome type 4 that combines the pronounced renal symptoms of other Bartter syndromes with sensorineural deafness (Birkenhäger et al., 2001). It is a 320 amino acid protein that contains two putative transmembrane domains followed by a long carboxy terminus. Truncations of barttin after position 72 left the function of ClC-K/barttin channels in heterologous expression systems almost unaffected (Scholl et al., 2006; Janssen et al., 2009). The interaction of Barttin with the ClC-K channels is proposed to be along the two transmembrane helices B and J, but further details about the interaction of channel and accessory subunit are still lacking (Figure 6C) (Tajima et al., 2007).

In mice and rats, ClC-K1, ClC-K2, and barttin are both distributed starting from the thin over the thick ascending duct of Henle's loop well into to the collecting duct (Vandewalle et al., 1997; Waldegger et al., 2002; Nissant et al., 2004). The two ClC-K isoforms, however, show differences in their expression along different parts of the nephron so that channels consisting of ClC-Ka and barttin are now thought to mediate the passage of Cl^−^ through the epithelium predominantly in the thin ascending limb while ClC-Kb/barttin is believed to be necessary for the reuptake of chloride in the thick ascending limb and sustaining the transepithelial potential that results in paracellular absorption of diverse cations including Ca^2+^ and Mg^2+^. ClC-K and barttin also exhibit abundant expression in the stria vascularis of the inner ear. ClC-K/barttin channels permit the basolateral efflux of Cl^−^ ions accumulated by the NKCC1 Na^+^-K^+^-Cl^−^ co-transporter which is necessary for the generation of the endocochlear potential.

Neither expression of ClC-Ka nor of ClC-Kb results in visible currents when the barttin subunit is lacking. Barttin exerts multiple functions on ClC-K channels. It supports the exit from the endoplasmic reticulum, stimulates insertion into and impairs removal of ClC-Ks from the surface membrane (Figure 6D) (Scholl et al., 2006). Moreover, barttin increases the ClC-K protein stability by stimulation of complex glycosylation (Figures 6E,F) (Hayama et al., 2003; Scholl et al., 2006; Janssen et al., 2009). ClC-Ka and ClC-Kb are not functional without the accessory subunit and are switched into a conducting state by association with barttin. Upon co-expression, ClC-Ka and ClC-Kb show time-independent, mostly non-rectifying currents (Janssen et al., 2009; Fischer et al., 2010). ClC-Ka/barttin channels are permanently open at voltages positive to −150 mV. The decay of the open probability at voltages negative to −150 mV results in a characteristic “hook” in macroscopic current-voltage plots. The single channel conductance of ClC-Ka is approximately 30 pS and thus significantly larger than for other CLC channels (Figure 3). So far, unitary properties of ClC-Kb have not been determined.

Several *CLCNKB* missense mutations have been found in patients with Bartter syndrome (Simon et al., 1997; Fukuyama et al., 2004). These mutations usually cause loss-of-channel function and are thus expected to reduce water reabsorption by affecting the cortico-medullary osmotic gradient. There is one report about a patient with combined mutations in *CLCNKA* and *CLCNKB*. As expected the patient suffers from severe renal salt wasting and sensorineural deafness which closely mimicked mutations in the *BSND* gene that also affect both chloride channels simultaneously (Schlingmann et al., 2004). One mutation—predicting T481S ClC-Kb—was found in a genetic screen of hypertensive cohorts. In heterologous expression systems it permits ClC-Kb channel opening even in the absence of barttin (Jeck et al., 2004; Sile et al., 2009). The mutation might increase salt and subsequently water reabsorption and thereby increase systemic blood pressure. Moreover, increasing chloride currents in the stria vascularis will augment the endocochlear potential and thereby lower the hearing threshold (Frey et al., 2006). Since reduced blood volume through increased water excretion may enhance renin secretion and upregulate the renin-angiotensin-aldosterone system (RAAS), loss-of-function of ClC-K/barttin channels might increase the risk of heart failure. Recently, a *CLCNKA* variant predicting R83G ClC-Ka was reported to result in loss-of-function of this channel and was suggested as risk factor for heart failure (Cappola et al., 2011). Other naturally occurring variants of ClC-Ka that increase systemic blood pressure following an increased NaCl load were linked to chronic, salt-dependent hypertension (Barlassina et al., 2007).

The important role of ClC-K/barttin channels in the regulation of the body's salt and water content makes these channels an important target for pharmacological intervention (Picollo et al., 2004; Liantonio et al., 2006, 2012; Imbrici et al., 2014). ClC-K/barttin activators might correct loss-of-function of channels carrying naturally occurring mutations associated with Bartter syndrome or idiopathic deafness. ClC-K/barttin channel blockers might serve as diuretics or as anti-hypertensive drugs. However, there are potential side effects that should be carefully investigated. Hearing appears to be much more sensitive to ClC-K channel activity (Riazuddin et al., 2009) than kidney function. Although the blood-labyrinth barrier shields the inner ear epithelia from the general circulation (Juhn and Rybak, 1981), several compounds such as salicylate, furosemide or aminoglycoside antibiotics readily affect inner ear function. Furthermore, a long term follow-up of patients suffering from Bartter syndrome showed persistent hyperreninemia, secondary hyperaldosteronism and development of proteinuria in many patients even if other symptoms are rather well controlled (Bettinelli et al., 2007). Possible side effects of pharmacological alterations in ClC-K function therefore have to be carefully tested and might even preclude a widespread clinical use.

Missense mutations that prevent barttin expression result in a very severe renal phenotype (Janssen et al., 2009; Fahlke and Fischer, 2010; Nomura et al., 2011) often with endstage renal failure in young adulthood. Since other *BSND* mutations that also abolish formation of functional ClC-K/barttin do preserve renal function it is tempting to speculate whether these channels exert additional functions in renal cells. Successful treatment of these rare cases might require a better understanding of the interaction of ClC-K with the subunit barttin. However, at present, it is not clear how barttin interacts with ClC-K channels, and it is also unclear whether this interaction can be targeted to modify channel function.

## Outlook

Anion channels exist in all cells of the human body and fulfill multiple physiological functions. The importance of these channels is highlighted by genetic variants associated to major diseases. Evolution has resulted in multiple anion transport protein families, with very different functions and physiological roles. The CLC family is currently the largest known gene family encoding anion channels and transporters, and many aspects of CLC channel function are already well understood. CLC channel function has been studied using whole-cell and single channel patch clamp recordings and fluorescence imaging. Three-dimensional structures from related proteins have provided detailed insights into the molecular architecture of these proteins. Using knock-out and knock-in animal models we can study the consequences of CLC functions on cellular processes.

CLC channels are functionally very different from voltage-gated cation channels, and many of these special properties are due to their evolution from transporters. Many secondary-active transporters can assume channel-like slippage modes (DeFelice and Goswami, 2007). However, the CLC family has undergone a rather strict separation between these modes into antiporters and channels without transporter activity. The molecular determinants of this differentiation are not clear. Whereas the replacement of a single amino acid (D136G) can convert one channel, ClC-1, into a channel with properties very similar to ClC-2, it has not yet been possible to transform a CLC channel into a CLC transporter. CLC channels have attracted a great deal of attention because of their unique double-barreled structure. We still do not understand the molecular functions and the physiological impact of this curious design. How do the two subunits communicate with each other, and what is the physiological advantage of having two more or less synchronized pores?

The newly predicted localization of ClC-1 in the central nervous system raises questions concerning potential roles of this channel in this organ system. It is still unclear how ClC-2 prevents neurodegeneration and which role mutant ClC-2 plays in central nervous hyperexcitability. ClC-Ka and -Kb are important pharmacological targets, however, no compound targeting these channels has yet entered clinical practice. The possible formation of hetero-dimeric CLC channels with properties greatly different from homo-dimeric channels also warrants further investigation. Although much knowledge exists about the chloride conductance of CLC channels, it might be worthwhile to study the permeation of bicarbonate as well. We also lack a thorough molecular understanding of the way accessory subunits such as barttin or GlialCAM modify CLC channels.

Studying the CLC family has always been driven by both, the curiosity about the biophysical properties and their physiological relevance. The last 25 years have provided the knowledge and the tools to address many of the remaining questions. This work will hopefully lead to a detailed understanding of the molecular physiology and pathophysiology of these channels and may improve the quality of life of many patients with rare or common diseases.

### Conflict of interest statement

The authors declare that the research was conducted in the absence of any commercial or financial relationships that could be construed as a potential conflict of interest.
